# Human Herpes Virus-6 (HHV-6) infectious encephalitis in an immunocompetent adult

**DOI:** 10.1016/j.idcr.2024.e01992

**Published:** 2024-05-26

**Authors:** Lisle Blackbourn, Sharjeel Ahmad, K’la Yuan, Manjari Uppu, Bassil Kherallah

**Affiliations:** a Department of Neurology, University of Illinois College of Medicine Peoria, Peoria, IL, United States; b OSF Illinois Neurological Institute, Peoria, IL, United States; c Department of Infectious Disease, University of Illinois College of Medicine Peoria, Peoria, IL, United States

**Keywords:** HHV-6, Encephalitis, Herpes Virus 6

## Abstract

Human herpesvirus 6 (HHV-6) is one of the most prevalent childhood viruses. HHV-6 reactivation in allogeneic hematopoietic cell transplant recipients and solid organ transplant recipients is well described in medical literature. We present a case of HHV-6 reactivation causing encephalitis, which is rare in immunocompetent adults.

## Introduction

HHV-6 is one of the most prevalent childhood viruses. It is acquired between the ages of 6 and 15 months, with some studies documenting up to 95 % infection rate in children [1]. Sources believe it accounts for 20 % of all acute fevers and children between the ages of 6 and 12 months old, most commonly causing exanthem subitum.

After initial infection, HHV-6 can establish latency within different sites in the body and is capable of reactivation later on in life. There are many documented cases of HHV-6 reactivation in allogeneic hematopoietic cell transplant recipients, with some sources citing up to a 40–50 % reactivation rate [2]. Patients can present with acute and severe complications related to meningoencephalitis and encephalomyelitis. The mortality rate is high in this population, especially without quick and definitive treatment.

Here we present a case of a 76-year-old immunocompetent female presenting with HHV-6 encephalitis.

## Case

Patient is a 76-year-old non-diabetic female with a history of seizure disorder (on oxcarbazepine 300 milligrams (mg) twice a day), hypothyroidism, hypertension, hyperlipidemia, and a past documented HSV-1 encephalitis infection in 2016 (causing multifocal cerebral and cerebellar encephalomalacia with gliosis of the posterior right cerebral hemisphere) who presented to an outlying hospital with altered mental status and syncopal episodes. Patient was found to be lethargic but able to follow basic commands and was weaker on the left side. Patient was transferred for further care due to concern for breakthrough seizures after being loaded with levetiracetam and started on maintenance 500 mg twice a day.

On arrival, the patient's vital signs were normal but she shortly after spiked a fever of 101 degrees Fahrenheit. Patient was lethargic but completely oriented. On physical exam, the patient had intact cranial nerves, intact sensation, normal reflexes but had dysarthria, generalized weakness (3/5) with worsening weakness in the left arm (1/5). Presentation was concerning at the time for possible subclinical versus overt seizures, ischemic stroke, and underlying infection. Patient was placed on continuous EEG, MRI brain with and without contrast was ordered, a full infectious workup was done including blood cultures and urinalysis. She was started on empiric coverage for infection that included vancomycin, ceftriaxone, ampicillin, and acyclovir given patient’s history of past encephalitis.

Continuous EEG monitoring was notable for frequent focal right posterior temporal nonconvulsive seizures with status epilepticus by electrographic criteria. Seizures were highly refractory, and eventually controlled by gradual initiation and titration of oxcarbazepine 600 mg twice a day, lacosamide 200 mg twice a day, levetiracetam 2000 mg twice a day, valproic acid 500 mg three times a day, and clonazepam 0.5 mg twice a day.

MRI brain was negative for any acute stroke but was notable for hyperemia in the right posterior temporal lobe cortex correlating well with seizure focus demonstrated on EEG. It did show multifocal cerebral and cerebellar encephalomalacia and severe volume loss and gliosis of the posterior right cerebral hemisphere from her past HSV-1 encephalitis infection that was unchanged from prior imaging.

Lumbar puncture was performed and showed a lymphocytic pleocytosis (11 cells with 96 % lymphocytes) with normal protein (34) and elevated glucose (77) and negative HSV PCR. CSF meningitis/encephalitis PCR panel (Mayo Clinic Lab) was positive for HHV-6, for which Infectious Disease was consulted. Due to the unexpected finding in an immunocompetent patient, CSF HHV-6 quantitative PCR was also tested and again positive, showing 2280 copies/mL. Blood microbial cell free DNA testing (mcfDNA) (Karius, Inc. Redwood City, CA) showed the presence of HHV-6B and *Streptococcus macedonicus*. *Streptococcus macedonicus* was deemed clinically insignificant. Antimicrobial regimen was simplified to Foscarnet once the above results were available. Baseline blood HHV-6 quantitative HHV-6 PCR showed 438,569 copies/mL. HHV-6 integration testing was consistent with chromosomal integration with a virus to cell ratio of 0.95.

Immunodeficiency screening including HIV testing, immunoglobulin testing, and CD4 count failed to reveal an immune deficiency state.

Patient remained in the hospital to complete a full 2-week course of treatment with Foscarnet. Her alertness level greatly improved and her strength testing improved to 4/5 in the upper extremities and was 3/5 in the lower extremities on discharge. Her dysarthria also resolved. She was weaned to levetiracetam 1000 mg twice a day, oxcarbazepine 600 mg twice a day, and lacosamide 200 mg twice a day on discharge to a rehabilitation facility. She was alert and fully oriented at the time of follow-up in the Infectious Diseases clinic 2 weeks after discharge from the hospital.

## Discussion

Our patient met the criteria for encephalitis by having encephalopathy with fever, seizures, and CSF pleocytosis, which are all indicative of CNS inflammation even in the absence of acute MRI brain changes [3]. Although our patient had a history of seizure disorder stemming from the multifocal cerebral and cerebellar encephalomalacia with gliosis caused by her past HSV-1 encephalitis infection, she was well controlled on oxcarbazepine and work-up was performed to look for a cause for nonconvulsive focal status epilepticus. It would be unusual for someone well controlled without seizure activity on 1 medication for at least 7 years to suddenly go into such severe status epilepticus that 5 seizure medications and sedative medication were needed to control the status epilepticus without a reason for breakthrough seizure activity. While it was noted that the patient had excellent compliance with medication, it cannot be fully ruled out that she did miss medications leading up to events. Patient’s fever also pointed to an infectious etiology. Originally, it was suspected to be a reactivation and reinfection of HSV-1. However, all testing resulted negative for HSV-1. The overall clinical picture was consistent with reactivation HHV-6 disease given her fever, CSF pleocytosis, encephalitis, status epilepticus, and improvement of seizure activity once started on foscarnet. It is also possible that patient’s improving neurological deficits were from a Todd’s palsy picture given resolution with cessation of the seizure activity.

Most cases of HHV-6 encephalitis have occurred in allogeneic hematopoietic cell transplant recipients, although it has also been reported rarely in solid organ transplant recipients and in immunocompetent individuals [4].

In a study involving children under 5 years of age with skin rash and negative for rubella and measles, 66 % were positive for HHV-6 IgM, and 91 % were positive for HHV-6 IgG [5]. The large majority of these children recovered with no neurological sequelae.

HHV-6 disease has been reported rarely in solid organ transplant recipients and in immunocompetent children and adults. A retrospective study documented 15 cases of HHV-6 infections in immunocompetent children ranging in age from 1 month to 7 years old, four of which were cases of HHV-6 meningoencephalitis [6]. All of the children had complete recovery without neurological deficits. HHV-6 reactivation causing encephalitis has been rarely documented in immunocompetent adults.

A literature search was completed on PubMed of HHV-6 encephalitis seen in immunocompetent adults can be seen in Table 1 with 15 cases being found in our search [4], [7], [8], [9], [10], [11], [12], [13], [14], [15], [16], [17], [18]. Seizures were present in 2/3rds of the cases with most patients being treated with acyclovir or ganciclovir. Patient outcomes ranged from complete recovery to death.

**Table 1:** Previously published cases of HHV-6 encephalitis in immunocompetent adults.

Year of Report	Age	Gender	Antiviral used	Seizures	Patient Outcome	Reference Number
2005	38	Female	Valganciclovir	Yes	Neurological deficits 1 year later	7
2005	66	Female	Acyclovir	No	Continued to have concentration difficulties, fatigue, and a slight tremor	7
2005	20	Female	Not reported	No	Not reported	7
2005	21	Female	Ganciclovir	Yes	Slight residual concentration deficit	8
2007	73	Female	Acyclovir	Yes	No residual sequelae	9
2008	34	Male	Ganciclovir --> Ganciclovir + Foscarnet	Yes	Nearly complete clinical recovery	10
2014	65	Female	Foscarnet --> Ganciclovir	Yes	Seizure free	11
2017	26	Male	Acyclovir	Yes	Symptom resolution	12
2018	29	Female	Acyclovir	No	No residual sequelae	13
2019	93	Female	None (went hospice)	Yes	Hospice	14
2021	63	Female	Ganciclovir	No	No recurrence of symptoms	4
2021	55	Female	Ganciclovir	Yes	Not reported	15
2021	46	Female	Ganciclovir	Yes	Death	16
2021	48	Male	Ganciclovir --> Valganciclovir	No	No residual sequelae	17
2022	26	Male	Ganciclovir --> Valganciclovir	Yes	Favorable clinical response	18

Chromosomally integrated human herpesvirus 6 (ciHHV-6) is a phenomenon where the HHV-6 genome is integrated into the host’s germ line genome [19]. The high virus to cell ratio of 0.95 in our patient is consistent with chromosomal integration. The high HHV-6 DNA load in patients with ciHHV-6 can sometimes lead to misdiagnosis due to the assumption of active HHV-6 infection but in this patient the clinical picture was consistent with HHV-6 infection with improvement on appropriate treatment. While elevated HHV-6 levels can be seen in settings such as alcoholism and use of immunosuppressives, clinical correlation is needed to establish a cause and effect relationship while looking for other possible etiologies for patient’s symptoms [20].

Currently, there are no Food and Drug Administration (FDA) approved therapies for HHV-6 infection [21]. Cidofovir, ganciclovir, and foscarnet have been used off-label. These medications however are not without side effects and complications, and therefore should be carefully considered for immunocompetent patients who may recover without the use of such medications. An unambiguous association between HHV-6 infection and disease is a major roadblock to the development of new antiviral therapies specific for HHV-6 infection.

## Conclusion

This case report highlights the rare presentation of HHV-6 encephalitis in an immunocompetent adult. While, there are no approved therapies solely for HHV-6 infection, cidofovir, ganciclovir, and foscarnet have been used for treatment of HHV-6 disease with noted clinical improvement.

## Declaration of Competing Interest

The authors declare that they have no known competing financial interests or personal relationships that could have appeared to influence the work reported in this paper.

## Consent

Written informed consent was obtained from the patient for publication of this case report and accompanying table. A copy of the written consent is available for review by the Editor-in-Chief of this journal on request.

## CRediT authorship contribution statement

**Lisle Blackbourn:** Writing – review & editing, Writing – original draft. **Sharjeel Ahmad:** Writing – review & editing. **K'la Yuan:** Writing – review & editing, Writing – original draft. **Manjari Uppu:** Writing – review & editing. **Bassil Kherallah:** Writing – review & editing.
